# Evaluation of Inhibitor-Resistant Real-Time PCR Methods for Diagnostics in Clinical and Environmental Samples

**DOI:** 10.1371/journal.pone.0073845

**Published:** 2013-09-09

**Authors:** Adrienne Trombley Hall, Ashley McKay Zovanyi, Deanna Rose Christensen, Jeffrey William Koehler, Timothy Devins Minogue

**Affiliations:** Diagnostic Systems Division, United States Army Medical Research Institute of Infectious Diseases, Fort Detrick, Maryland, United States of America; Naval Research Laboratory, United States of America

## Abstract

Polymerase chain reaction (PCR) is commonly used for pathogen detection in clinical and environmental samples. These sample matrices often contain inhibitors of PCR, which is a primary reason for sample processing; however, the purification process is highly inefficient, becoming unacceptable at lower signature concentrations. One potential solution is direct PCR assessment without sample processing. Here, we evaluated nine inhibitor-resistant PCR reagents for direct detection of *Francisella tularensis* in seven different clinical and environmental samples using an established real-time PCR assay to assess ability to overcome PCR inhibition. While several of these reagents were designed for standard PCR, the described inhibitor resistant properties (ex. Omni Klentaq can amplify target DNA samples of up to 20% whole blood or soil) led to our evaluation with real-time PCR. A preliminary limit of detection (LOD) was determined for each chemistry in whole blood and buffer, and LODs (20 replicates) were determined for the top five chemistries in each matrix (buffer, whole blood, sputum, stool, swab, soil, and sand). Not surprisingly, no single chemistry performed the best across all of the different matrices evaluated. For instance, Phusion Blood Direct PCR Kit, Phire Hot Start DNA polymerase, and Phire Hot Start DNA polymerase with STR Boost performed best for direct detection in whole blood while Phire Hot Start DNA polymerase with STR Boost were the only reagents to yield an LOD in the femtogram range for soil. Although not the best performer across all matrices, KAPA Blood PCR kit produced the most consistent results among the various conditions assessed. Overall, while these inhibitor resistant reagents show promise for direct amplification of complex samples by real-time PCR, the amount of template required for detection would not be in a clinically relevant range for most matrices.

## Introduction

Real-time PCR assays are commonly used for the rapid detection of biological organisms in complex environmental and clinical matrices [1–8]. Rapid and specific diagnosis of infection for emerging agents is critical for applying the appropriate countermeasures as time to detection and treatment can impact prognosis and pathogen containment. These types of PCR assays work through primer-dependent amplification of specific nucleic acid targets and probe-based fluorescence detection of the target amplicon [9]. One such probe-based technology is TaqMan. This assay relies on *Taq* DNA polymerase’s inherent 5’–3’ exonuclease function to digest a FRET-based probe in the DNA polymerization reaction, releasing the probe-bound fluorophore and cognate quencher. The unquenched fluorophore is detected, and relative fluorescence is translatable to the relative amount of amplicon. TaqMan and other probe-based assays require all components of the PCR reaction to be accessible and perform optimally for efficient detection and diagnosis.

A variety of different inhibitors inherent to clinical and environmental matrices can negatively impact PCR sensitivity and accurate pathogen detection. Interference with cell lysis, sequestration or degradation of nucleic acids, and hindrance of polymerase activity are all common mechanisms of PCR inhibition [10]. Heme present in blood can block the polymerase’s active site [11] while proteases can degrade the polymerase. Other inhibitors include complex polysaccharides [12], bilirubin and bile salts [13] found in stool, as well as humic compounds in soils and sediments [14]. Options for preventing or lowering the amount of PCR inhibition include sample processing and/or decreasing the amount of sample matrix, thereby removing or diluting matrix-derived inhibitors [15–17]. However, both of these strategies can negatively impact target nucleic acid concentration. Strategies to overcome PCR inhibition include adding different components to the PCR reaction. For example, bovine serum albumin [18,19], betaine [18], or proteinase inhibitors [18] have been used to reduce PCR inhibition. Other methods of inhibitor relief focus on modification of DNA polymerase such as deletion of the N-terminal portion of the DNA polymerase [20].

Sample processing prior to PCR detection serves a number of purposes including: concentration of the target template, removal of inhibitory factors, and release of the purified target nucleic acid. However, extraction technologies are not 100% efficient; therefore, yields from sample processing are always less than the original nucleic acid input [15]. This is a significant issue if the target nucleic acids are present at low concentrations as they are in most clinical and environmental samples. Furthermore, some inhibitors can co-elute following purification and induce false negatives. For instance, false-negative tests were observed after several different types of purification methods followed by PCR detection for hepatitis B virus [21]. False negatives, especially for highly pathogenic agents, have significant consequences for both treatment and rapid containment in the event of an infection. Direct PCR detection from complex samples is a potential solution for yield loss with the added benefit of a decreased time-to-answer for etiologic agent identification. Utilization of inhibitor-resistant PCR reagents could increase the sensitivity of existing real-time PCR assays through direct amplification of nucleic acid targets at non-permissive concentrations of matrix inhibitors.

The objective of this research was to systematically evaluate inhibitor-resistant, commercially available buffers and polymerases to determine the efficacy in overcoming PCR inhibitors found in buffer, whole blood (WB), sputum, stool, swab, sand, and soil. Nine different chemistries or polymerases were acquired and tested independently and in combination using a well characterized real-time PCR assay for detection of *Francisella tularensis* [4]. Different concentrations of purified, genomic DNA from *F. tularensis* SCHU S4 were spiked into dilutions of the seven different matrices with LODs determined using real-time PCR to identify the lowest amount of template detectable at the highest concentration of sample matrix. Not surprisingly, different chemistries worked better for different types of inhibitory matrices.

## Materials and Methods

### Bacterial DNA

*F. tularensis* SCHU S4 genomic DNA (Critical Reagents Program) was used for these studies. DNA concentrations were measured using a Beckman DU640 spectrophotometer (Beckman Coulter, Inc., Brea, CA) and/or Nanodrop (ThermoScientific, Wilmington, DE). DNA was prepared using two different dilution series in matrix comprising 10 pg, 1 pg, 0.1 pg, 0.05 pg, and 0.01 pg as well as 2 pg, 0.2pg, 0.02 pg, 0.01 pg, and 0.002 pg, and each chemistry was evaluated with both dilution series. Based on the sequence of *F. tularensis* SCHU S4 (GenBank NC_006570.2), one genome equivalent (GE) is approximately 1.94 fg, so 2 fg DNA contains ~1 GE.

### Sample Matrices

Seven different sample matrices were evaluated for PCR inhibition including Dulbecco’s phosphate-buffered saline [PBS, (Life Technologies, Grand Isle, NY)], WB (collected in K2 EDTA BD Vacutainer tubes), sputum (no known additives), swab, stool, sand, and soil. WB, sputum, and stool were purchased from Bioreclamation (Westbury, NY) while sand and soil were acquired from the National Institute of Standards and Technology (Germantown, MD). Five g of sand, soil, or stool were resuspended in 50 ml of PBS containing 0.05% Tween-20 (Sigma-Aldrich, St. Louis, MO) to generate a 10% (w/v) stock. For the sputum sample, 0.15% (w/v) dithiothreitol (Sigma-Aldrich) was added as a mucolytic agent in order to reduce viscosity. Swab matrix was generated by swabbing both laboratory countertops and the floor and then soaking the swabs in PBS (1 ml per swab) with agitation. Swabs were removed, and the resulting mixture of swab material, laboratory particulates, and PBS were used in subsequent experiments. Matrix dilutions were generated to the indicated percent matrix concentration (v/v) with molecular biology grade water (Eppendorf, Hauppauge, NY), taking into account the further dilution of the added SCHU S4 DNA. The final concentration of matrix is reported as the percent matrix (v/v) within the PCR reaction. The *F. tularensis* genomic DNA concentration was represented as total amount of genomic DNA per PCR reaction.

### PCR Reaction Conditions

*F. tularensis* PCR master mix (FT-MM) for real-time PCR targeted the *tul4* gene and was prepared as previously described [4]. FT-MM contained 1x PCR buffer (BioFire, Salt Lake City, UT) and 0.8 units Platinum *Taq* DNA polymerase (Life Technologies) per reaction. This assay has a LOD of 50 fg (~25 genome equivalents) for 60 out of 60 replicates using PBS as the sample matrix [4]. Nine different experimental chemistries (see Table 1 for component descriptions) were assessed in these studies. While these reagents were not designed for real-time PCR, the components are resistant to PCR inhibitors. Three different PCR buffers designed for amplification in inhibitory samples were used, including PCRboost (Biomatrica, San Diego, CA), STRboost (Biomatrica), and Ampdirect (Rockland Immunochemicals, Gilbertsville, PA). Phusion Blood Direct PCR Kit (New England Biolabs, Ipswich, MA), Hemo KlenTaq (New England Biolabs), KAPA Blood PCR Kit (KAPA Biosystems, Woburn, MA), and Omni Klentaq (DNA Polymerase Technology, St. Louis, MO) were all designed for PCR amplification in blood and other inhibitory samples. Phire Hot Start DNA Polymerase (New England Biolabs) was an enhanced DNA polymerase, and Terra PCR Direct Polymerase Mix (Clontech, Mountain View, CA) was used for use with tissues and crude samples.

**Table 1:** Description of each chemistry tested with *F. tularensis*.

**Name of chemistry**	**Manufacturer**	**Polymerase/buffer**	**Product description**
Ampdirect	Rockland Immunochemicals	Buffer	Neutralizes inhibitory substances in human blood for direct DNA amplification.
Phire Hot Start DNA Polymerase	Finnzymes/New England Biolabs	Buffer plus polymerase	The enhanced polymerase enables short extension times, improved yields, and amplifies long DNA fragments.
Omni Klentaq	DNA Polymerase Technology	Polymerase	Overcomes inhibitors in 20% whole blood or crude soil samples.
Phusion Blood Direct PCR Kit	Finnzymes/New England Biolabs	Buffer plus polymerase	Designed to perform PCR directly from whole blood with no prior DNA extraction.
KAPA Blood PCR Kit	KAPA Biosystems	Buffer plus polymerase	Amplification of DNA fragments directly from whole blood.
Hemo KlenTaq	New England Biolabs	Buffer plus polymerase	N-terminal (280 amino acid) truncation and mutations allow resistance to inhibitors for direct amplification in whole blood.
PCRboost	Biomatrica	Buffer	Improves amplification of low copy transcripts, degraded, trace, or inhibitory samples.
STRboost	Biomatrica	Buffer	For use with limited, degraded, and blood samples.
Terra PCR Direct Polymerase Mix	Clontech	Buffer plus polymerase	Direct amplification from tissue samples, crude extracts, and dirty templates.

Inhibitor-resistant chemistries were used according to the manufacturer’s specifications for the initial evaluations but scaled down to a 20 µl reaction for comparability to the FT-MM. For the chemistries only containing reaction buffer (Ampdirect, PCR boost, and STR boost), 0.8 units Platinum *Taq* DNA polymerase (Life Technologies) were added per reaction in addition to the experimental chemistry. Preliminary data demonstrated that the fluorescent signal in the real-time PCR assays was decreased without the addition of Platinum *Taq*; therefore, 0.8 units of polymerase were included in all subsequent PCR reactions. For all reactions, 5 µl sample (DNA diluted in matrix) was added to 15 µl master mix, incorporating an additional 1:4 dilution of matrix. The percentage of each matrix in the final reaction volume is presented. For example, 5 µl of undiluted WB would be reported as 25% matrix.

All reactions were evaluated by real-time PCR on the R.A.P.I.D. platform (BioFire). Negative template controls containing water and master mix were run with each chemistry. Cycling conditions for all chemistries except for Terra PCR Direct Polymerase Mix (Terra PCR Direct) included a 2 min denaturation step at 95°C and 45 cycles of amplification at 95°C for 1 sec and 60°C for 20 sec. A single fluorescence read was taken at the end of each 60°C amplification cycle. For Terra PCR Direct, cycling conditions included a 2 min denaturation step at 98°C and 40 cycles of amplification at 98°C for 10 sec, 60°C for 15 sec, and 1 min at 68°C. A single fluorescence read was taken at the end of each 68°C cycle. A positive PCR reaction was defined as having a fluorescence signal that crosses the threshold (Cq) at <40 cycles. Control experiments in PBS were run in parallel with the inhibited sample to ensure template integrity and the proper PCR amplification. Overall PCR kinetics were rated based on a qualitative 1 to 5 scale as represented in Table 2. Qualitative metrics were applied across the target DNA dilution series for each percent matrix.

**Table 2:** Description of the real-time PCR curve scoring.

Qualitative score	Positive characteristics	Negative characteristics
1	a. Sample detection – software based Cq determination	a. Background fluorescence continuously decreases below starting levels with jagged readings (>1 RFU change)
		b. Amplification curves are not sigmoidal
		c. Endpoint fluorescence is inconsistent
		d. Replicate curves are not parallel, and the Cq values vary (>1 Cq difference)
2	a. All characteristics in 1 b. Amplification curves are sigmoidal	a. Background fluorescence continuously decreases below starting levels (>1 RFU change)
		b. Endpoint fluorescence is inconsistent
		c. Replicate curves are not parallel but maintain similar Cq values (1-2 Cq difference)
3	a. All characteristics in 2	a. Background fluorescence is rough with jagged readings
	b. End point fluorescence remains consistent	b. Replicate curves have some separation with similar Cq values (<1 Cq difference)
4	a. All characteristics in 3	a. Background fluorescence has some inconsistency
	b. Replicate curves are parallel with very similar Cq values (<1 Cq difference)	
5	a. All characteristics in 4	
	b. Background fluorescence remains smooth and consistent (<1 RFU difference)	

### Preliminary LOD Estimation and Confirmation

Preliminary LOD estimations were conducted with the different chemistries evaluated using our current methods accepted by the Commission on Office Laboratory Accreditation (COLA) for the DoD Clinical Laboratory Improvement Program (CLIP) requirements for a laboratory developed test (LDT). Different amounts of target nucleic acids were added to dilutions of matrix in order to identify the lowest amount of DNA detected in the highest amount of matrix. These preliminary LODs were confirmed using 20 replicates at the determined concentration across duplicate samples. A minimum of 19 of 20 replicates yielding positive results was required for LOD confirmation. Based on binomial sampling statistics, 19 of 20 replicates represents 82% probability for success at 85% confidence with a corollary calculated 95% sensitivity (true positives/true positives + false negatives). Several chemistries yielded 20 of 20 positive replicates representing an 87% probability of success at a 90% confidence based on the same statistics.

## Results

### PCR Chemistries

In this study, we sought to systematically evaluate several of the commercially available PCR chemistries and enzyme sets for real-time PCR use in the presence of inhibitory concentrations of diverse sample matrices, specifically: PBS, WB, sputum, swab, stool, sand, and soil. While these products were designed for standard PCR, the inhibitor resistant properties of these reagents made an evaluation using real-time PCR attractive: based on the manufacturers’ documentation, each chemistry/enzyme has specific characteristics and advantages for discrete uses (Table 1). Additionally, most of these products are amenable to our lab’s diagnostic and biosurveillance mission. All technologies in Table 1 were included into the overall experimental design to determine empirically if there were additional clinical and environmental applications beyond that described by the manufacturer.

### Baseline Matrix Inhibition Determination with FT-MM

The inhibitory effects of each matrix were initially evaluated using our standard FT-MM assay in a preliminary LOD determination (Table 3). Serial dilutions of *F. tularensis* genomic DNA were used with dilutions of each clinical and environmental matrix. Complete inhibition of PCR was observed with concentrations of 2.5% WB and sand as well as soil at 0.05%; there was sporadic, nonlinear inhibition in sputum (Table 3). The swab matrix showed no observable inhibition with the Platinum *Taq* master mix (FT-MM).

**Table 3:** Preliminary LOD determination (2/2) using FT-MM across each matrix.

**Matrix**	**Percent matrix**	**LOD**
Whole blood	2.5%	ND
	0.5%	0.02 pg
	0.25%	0.01 pg
	0.05%	0.01 pg
Sputum	2.5%	10 pg
	0.5%	0.2 pg
	0.25%	1 pg
	0.05%	0.02 pg
Swab	25%	0.01 pg
	5%	0.01 pg
Stool	2.5%	0.01 pg
	0.5%	0.002 pg
Soil	0.25%	ND
	0.05%	ND
	0.025%	0.01 pg
	0.005%	0.01 pg
Sand	2.5%	ND
	0.5%	0.02 pg
	0.25%	0.01 pg
	0.05%	0.01 pg

ND, not detected at any DNA concentration

### Optimization of Standard PCR Reagents for Real-Time PCR

Omni Klentaq, Hemo KlenTaq, and Terra PCR Direct all have an N-terminal truncation resulting in a Klenow fragment, a known mechanism for inducing a more inhibitor-resistant DNA polymerase [20]. Phusion and Phire are modified *Pyrococcus* -like enzymes that the lack 5’–3’ exonuclease activity, which is required for probe digestion and fluorophore detection. Of the polymerases tested here, only KAPA maintains an intact 5’–3’ exonuclease activity. We hypothesized that addition of a *Taq* DNA polymerase with 5’–3’ exonuclease activity might mitigate the potential absence of this activity in the inhibitor-resistant enzymes. Experiments with the inhibitor-resistant polymerases in buffer, performed with and without intact Platinum *Taq*, showed an improvement in performance when Platinum *Taq* was added (Table 4). Specifically, the preliminary LOD for Phire improved from 10 pg DNA to 0.05 pg DNA. While no DNA was detected in Terra PCR Direct without Platinum *Taq*, 100 pg DNA was detected upon addition. While the improvement with Terra PCR Direct was marginal, it remained below ranges applicable for clinical and environmental detection. As a result, all subsequent experiments contained Platinum *Taq* polymerase.

**Table 4:** Comparison of inhibitor-resistant polymerases with and without *Taq* DNA polymerase addition in buffer.

	**Without added *Taq***				**With added *Taq***		
**Chemistry**	**25% buffer**	**Average C_T_**	**Score**		**25% buffer**	**Average C_T_**	**Score**
Phire	10 pg	31.74	2		0.050 pg	36.42	4
Omni Klentaq	0.01 pg	35.63	4		0.050 pg	31.75	3
Phusion	0.01 pg	38.1	4		0.01 pg	37.73	5
KAPA	0.01 pg	32.05	4		0.01 pg	34.74	5
Hemo KlenTaq	0.01 pg	37.65	1		0.01 pg	35.04	2
Terra PCR Direct	ND	-	0		100 pg	33.66	2

See Table 2 for real-time PCR Score description

Cq values represent average Cq value across two replicates

ND, not detected at any DNA concentration

Surprisingly, we did observe a fluorescence signal when these enzymes were used without *Taq*. While this was unexpected since there is no 5’–3’ exonuclease activity, we hypothesized that the polymerase was priming off of the probe annealed to the template, and the strong 3’–5’ exonuclease activity was hydrolyzing the 3’ end of the probe to generate the observed signal. Data generated using gel-purified amplicon and excess probe without any primers suggest this hypothesis is correct as fluorescence was observed in template concentration-dependent fashion (data not shown).

Of note, these and almost all subsequent experiments with inhibitor-resistant PCR reagents showed less than ideal real-time PCR kinetics with amplification of the target, i.e. non-sigmoidal amplification, wavering baseline, and inconsistent endpoint fluorescence (see Figure S1A for an example). Because the qualitative aspect and visualization of the real-time PCR kinetics increases confidence in software-based adjudication of Cq values, we scored reactions using a qualitative 1 to 5 scale with 5 being ideal and 1 being unacceptable (see Table 2 for curve characteristics).

### Inhibitor-resistant Chemistry Down-selection

An initial down-selection based on preliminary LODs (2/2 replicates) was conducted using each chemistry with buffer and WB (Table 5). Buffer matrix represented performance of the chemistry or polymerase without inhibitors while WB was used to determine inhibition relief in a common clinical matrix. Experiments in buffer showed that the different chemistries or polymerases did not negatively impact PCR detection of *F. tularensis* DNA compared to the FT-MM control. However, most inhibitor-resistant reagents mitigated WB inhibition showing amplification of the target at a higher percent matrix than FT-MM. Specifically, detection of *F. tularensis* DNA in WB with Ampdirect, Phire, Phusion, and KAPA performed adequately at relatively high concentrations of matrix (0.5% to 2.5% WB per reaction), detecting the nucleic acid at clinically relevant concentrations (i.e., 0.05 pg or less) which wasn’t detectable using the FT-MM (Table 5). Inconsistent, non-intuitive data were noted for several chemistries, i.e., Ampdirect required 10 pg DNA for detection at 0.25% WB yet 0.01 pg DNA was detected in 0.5% WB. This effect could be a result of a low replicate number (two replicates) or differential amount of inhibitors within discrete reactions. Inhibitor-resistant reagents yielded lower quality metrics for WB compared to buffer (Table 5). Only PCRboost and STRboost, where reactions contained the same Platinum *Taq* as the FT-MM, demonstrated similar PCR kinetics as buffer (Table 5). Of note with WB, the incorporation of STRboost improved the quality of the real-time PCR curves (Figure S1 for a comparison).

**Table 5:** Impact of inhibitor-resistant reagents on detection and qualitative real-time PCR characteristics.

	**Buffer**			**Whole blood**				
**Chemistry**	**25%**	**Score**		**2.5%**	**0.5%**	**0.25%**	**0.05%**	**Score (matrix) %)**
Ampdirect	0.01 pg	5		ND	0.01 pg	10 pg	0.2pg	1 (0.5%)
Phire	0.050 pg	4		0.05 pg	0.01 pg	0.050 pg	0.01 pg	2 (0.5%)
Omni Klentaq	0.050 pg	2		10 pg	ND	ND	0.2 pg	1 (0.05%)
Phusion	0.01 pg	5		0.01 pg	0.01 pg	0.050 pg	0.01 pg	2 (0.5%)
KAPA	0.01 pg	3		0.1 pg	0.01 pg	0.050 pg	0.02 pg	1 (0.5%)
Hemo KlenTaq	0.050 pg	3		ND	0.002 pg	ND	0.01 pg	1 (0.5%)
PCRboost	0.01 pg	5		ND	ND	1 pg	0.2 pg	3 (0.05%)
STRboost	0.01 pg	5		ND	ND	0.1 pg	0.02 pg	3 (0.05%)
Terra PCR Direct	0.01 pg	2		ND	ND	ND	ND	ND
FT-MM	0.01 pg	5		ND	ND	ND	0.01 pg	3 (0.05%)
Phire plus Ampdirect	0.01 pg	5		0.050 pg	0.01 pg	0.01 pg	0.002 pg	2 (0.5%)
Phire plus STRboost	0.01 pg	5		0.1 pg	0.02 pg	0.050 pg	0.02 pg	3 (0.5%)
Phire plus PCRboost	0.01 pg	4		0.050 pg	0.02 pg	0.050 pg	0.02 pg	2 (0.05%)

See Table 2 for real-time PCR Score description

2/2 replicates had to be positive for the limit of detection call

ND, not detected at any DNA concentration

Zhang et al. showed that the combination of an inhibitor-resistant cocktail (trehalose sugar, nonionic detergent, carnitine, and heparin) coupled with an inhibitor resistant DNA polymerase improved PCR detection under non-permissive conditions when compared to the polymerase alone [22]. We combined reagents that had a separate polymerase with reagents that were solely buffer-based and tested for inhibition relief to determine if combinations of various chemistries and polymerases would improve performance (Table 5 and data not shown). Reagents that were buffer only (Ampdirect, STRboost, and PCRboost) were combined in matrix fashion with the polymerases Phire, Omni Klentaq, or Hemo KlenTaq. Only Phire combined with Ampdirect or STRboost showed similar or improved performance for mitigating WB inhibition. All combinations of Omni Klentaq and Hemo KlenTaq showed similar or poorer inhibition relief when matrixed across inhibitor-resistant buffers (data not shown). The following chemistries were selected based on the collective data from Table 5 for further evaluation: Phusion, Phire, KAPA, Ampdirect plus Phire, and STRboost plus Phire.

### Estimation and Confirmation of LOD for Different Matrices

The five down-selected chemistries were evaluated for inhibition relief in the clinical matrices sputum and stool as well as environmental matrices soil and sand for an estimated LOD (Table 6). No one chemistry or combination of chemistries performed best across all respective matrices. For instance, Phire was the best performer in sputum, detecting 0.05 pg in the highest concentration of matrix; however, it performed the worst with stool. In contrast, Phire plus Ampdirect performed well in 5% stool, detecting 0.01 pg of the DNA target, while requiring 1 pg of template for detection in 2.5% sputum. Overall, KAPA yielded the most consistent detection across all of the sample matrices in LOD estimations detecting 0.1 pg in 2.5% sputum, 0.01 pg in 5% stool, 0.02 pg in 0.05% soil, and 0.01 pg in 2.5% sand (Table 6).

**Table 6:** Preliminary estimation of LOD down-selected inhibitor-resistant reagents for sputum, stool, soil, sand, and swab.

					**Phire plus**	**Phire plus**
**Matrix**	**% matrix**	**Phusion**	**Phire**	**KAPA**	**Ampdirect**	**STRboost**
Sputum	2.50%	ND	0.050 pg	0.1 pg	1 pg	1 pg
	0.50%	0.2 pg	0.01 pg	0.02 pg	0.2 pg	0.2 pg
Stool	25%	ND	ND	ND	ND	ND
	5%	0.02 pg	0.1 pg	0.01 pg	0.01 pg	0.01 pg
	2.50%	0.01 pg	0.050 pg	0.01 pg	0.01 pg	0.01 pg
	0.50%	0.01 pg	0.002 pg	0.01 pg	0.002 pg	0.01 pg
Soil	0.25%	ND	ND	ND	ND	ND
	0.05%	0.01 pg	0.2 pg	0.02 pg	0.01 pg	0.2 pg
Sand	2.50%	0.050 pg	0.050 pg	0.01 pg	1 pg	ND
	0.50%	0.2 pg	0.01 pg	0.02 pg	0.02 pg	2 pg
	0.25%	0.01 pg	0.01 pg	0.01 pg	0.050 pg	1 pg
	0.05%	0.2 pg	0.01 pg	0.01 pg	0.01 pg	0.01 pg
Swab	25%	0.01 pg	1 pg	0.01 pg	0.01 pg	0.050 pg
	5%	0.01 pg	0.01 pg	0.002 pg	0.01 pg	0.02 pg

2/2 replicates had to be positive for the limit of detection call

ND, not detected at any DNA concentration

Confirmation of LOD studies (20 replicates at the preliminary LOD) continued this trend of consistent performance with KAPA and matrix-specific performance with other inhibitor-resistant reagents (Table 7). Specifically, KAPA reactions yielded positive PCR results for 0.02-0.2 pg *F. tularensis* DNA in 0.5% WB and sputum, 0.05% soil, 5% stool, 2.5% sand, and 25% swab matrices. This did not equate with the best performer for each matrix. Both Phusion and Phire alone detected 0.2 pg target nucleic acid in 20 of 20 replicates at 0.5% WB with lower overall Cq values compared to KAPA, which only detected 19 of 20 replicates. Similarly, the combination of Phire with Ampdirect or STRboost produced the best results in stool, detecting 20 of 20 replicates with the lowest Cq values. KAPA as well as Phire in combination with either Ampdirect or STRboost yielded the lowest LODs for soil. Lastly, KAPA was the best performer for sand, yielding an LOD of 0.05 pg in 2.5% matrix while Phire had the lowest LOD of 0.02 pg in 25% swab. In terms of reproducibility, only blood appeared to be problematic for the reagents tested yielding CVs in the double digit range with the exception of Phire plus STRboost, which produced a CV of 1.72% across replicates. Inconsistencies in confirmed LODs (Table 7) compared to those determined in Table 6 estimation are a result of the initial confirmation not yielding a minimum of 19 of 20 positive replicates. In these cases, the next highest concentration in the dilution series of *F. tularensis* DNA was used and LOD confirmation repeated.

**Table 7:** Limits of detection (20/20) for each matrix.

**Chemistry**	**Matrix**	**% matrix**	**LOD**	**Cq ± STDEV**	**CV**
Phusion	Whole blood	0.5%	0.2 pg	28.11 ± 3.32	11.81
	Sputum	0.5%	0.2 pg	35.11 ± 0.38	1.09
	Stool	5%	0.02 pg	37.18 ± 1.72*	4.62
	Soil	0.05%	2 pg	32.08 ±1.22	3.82
	Sand	2.5%	0.1 pg	35.07 ± 0.42	1.20
	Swab	25%	0.05 pg	34.19 ± 0.33	0.97
Phire	Whole blood	0.5%	0.2 pg	28.90 ±3.70	12.82
	Sputum	0.5%	0.2 pg	36.80 ± 1.01	2.74
	Stool	2.5%	0.05 pg	35.25 ± 0.55	1.56
	Soil	0.05%	2 pg	ND for 15/20	-
	Sand	2.5%	0.1 pg	37.02 ± 1.45	3.91
	Swab	5%	0.02 pg	35.08 ± 0.59	1.67
KAPA	Whole blood	0.5%	0.2 pg	31.28 ± 3.51*	11.22
	Sputum	0.5%	0.2 pg	33.92 ± 0.19	0.57
	Stool	5%	0.02 pg	34.61 ± 0.62*	1.78
	Soil	0.05%	0.2 pg	32.60 ± 0.31	0.96
	Sand	2.5%	0.05 pg	35.09 ± 0.46	1.32
	Swab	25%	0.05 pg	33.31 ± 0.26	0.79
Phire plus	Whole blood	0.5%	0.2 pg	23.05 ± 7.73*	33.54
Ampdirect	Sputum	0.5%	0.2 pg	35.33 ± 0.68	1.94
	Stool	5%	0.02 pg	35.20 ± 1.77	5.02
	Soil	0.05%	0.2 pg	32.36 ± 0.51	1.59
	Sand	0.05%	0.2 pg	34.13 ± 0.60	1.77
	Swab	25%	0.1 pg	33.06 ± 0.41	1.24
Phire plus	Whole blood	0.5%	0.2 pg	32.28 ± 0.56	1.72
STRboost	Sputum	0.5%	0.2 pg	34.40 ± 0.67*	1.94
	Stool	5%	0.02 pg	35.00 ± 0.91	2.60
	Soil	0.05%	0.2 pg	30.03 ± 0.36	1.20
	Sand	0.5%	0.2 pg	34.18 ± 0.81	2.38
	Swab	25%	0.05 pg	33.60 ± 0.39	1.17

ND, not detectable

* 19/20 replicates were positive

## Discussion

The principal reason nucleic acids are purified from clinical and environmental samples prior to PCR is to remove a significant amount of PCR inhibitors in these samples. This purification process can be highly inefficient as we previously observed with DNA purification using chaotropic lysis and silica-based nucleic acid binding [15]. While purification would be appropriate for signatures at sufficient concentrations, this approach might be suboptimal for rare nucleic acid signatures, especially for pathogen detection. Also, specific extraction processes are typically applied based on sample matrix and/ target organism. This study sought to investigate direct PCR assessment in clinical and environmental samples using inhibitor-resistant PCR reagents as a mechanism to increase the sensitivity of real-time PCR assays as well as remove sample-specific sample preparation.

Given the manufacturer-described functionality, reagents designed for PCR amplification directly in clinical and environmental samples were selected and evaluated. Generally, the polymerases tested did not generate a sufficient fluorescence signal when used alone, likely due to the absence of 5’–3’ exonuclease activity in all of the polymerases except for the KAPA polymerase. This 5’–3’ exonuclease function is a critical component for probe-based hydrolysis [23]. Inclusion of *Taq* polymerase, having this exonuclease activity, improved probe hydrolysis detection. Interestingly, all but one of the inhibitor-resistant enzymes produced some baseline target-specific detection without the addition of native Taq polymerase. This is likely due to these polymerases priming from the probes bount to the target template and hydrolyzing the 3’ end of the probe for fluorescence signal generation.

Even with the added *Taq*, several of the chemistries tested did not perform as well as expected based on previous studies [20] or advertised functionality. For instance, Omni Klentaq was designed and tested to perform PCR in samples of up to 20% blood or soil [20], yet using our *F. tularensis* assay, we had inconsistent results across different matrix concentrations. Hemo KlenTaq, a Klenow fragment derivation, showed similar results. The primary reason for this could be these polymerases were designed for PCR and SYBR green real-time PCR rather than probe-based detection [20]. However, Zhang et al. demonstrated that coupling Omni Klentaq, which has an N-terminal truncation, and OmniTaq, which has this activity, with a cocktail of trehalose sugar, nonionic detergent, carnitine, and heparin allowed for robust probe- based detection [22]. This PCR enhancer cocktail was not available at the time of our study. However, we observed improved qualitative PCR kinetics with Phire and the PCR enhancer STRboost (a trehalose sugar-based reagent), suggesting the combination of inhibitor-resistant polymerase, functional 5’–3’ exonuclease activity, and trehalose sugar might be a viable starting point for the development of inhibitor-resistant cocktails.

Throughout these experiments, no single chemistry was superior across all of the matrices evaluated, similar to previous studies of inhibitor-resistant PCR formulas [24]. For detection in soil, Phire plus STR Boost performed best. For direct detection in whole blood, Phusion, Phire, and Phire plus STR Boost performed best. KAPA with Platinum *Taq* produced the most consistent results based on both qualitative and quantitative data within this study. However, all the reagents assessed for analytical LODs detected signatures at or below relevant template concentration, i.e., femtogram range. None of these reagents would be directly applicable as necessary dilution of all sample matrixes with the expectation of swabs would reduce the functional template concentration below relevance for detection. For example, a 0.5% dilution of WB, as identified in our confirmation of LOD studies as overcoming PCR inhibition, would be 0.1 µl WB in the PCR reaction. The confirmed LOD for our assay in 0.5% WB was 0.2 pg; *F. tularensis* would have to be at 2 ng/ml or ~10^6^ GE/ml WB in order to be consistently detectable. This approach would not applicable for clinical diagnostic detection at this required dilution and amount of available template in the sample.

Overall, these studies show promise and potentially a path forward for pathogen detection by real-time PCR directly in a clinical or environmental sample and removal of sample processing. Future studies would be required to further optimize these and other inhibitor-resistant PCR reagents such that LODs would be within relevant ranges as well as assay optimization for intact organism detection.

## Supporting Information

Figure S1 **PCR kinetics and endpoint fluorescence comparison for Phire Hot Start and STRboost plus Phire Hot Start Polymerase.** Both master mixes were tested with serial dilutions of DNA in 0.5% whole blood. On the y-axis (fluorescence), the range is -4.5 to 3 with increments of 0.5. The x-axis (cycle number) is 0 to 45 with increments of 5. (A) Phire Hot Start DNA polymerase master mix generated less than ideal real-time PCR kinetics and endpoint fluorescence. (B) When Phire Hot Start Polymerase was used with STRboost, the real-time PCR kinetics and endpoint fluorescence was greatly improved. The y-axis (fluorescence) ranges from -0.5 to 5.5 with increments of 0.5. The x-axis (cycle number) is 0 to 45 with increments of 5.
(TIF)
