# Supplementary figures and images for: Evaluation of Inhibitor-Resistant Real-Time PCR Methods for Diagnostics in Clinical and Environmental Samples
Source: PLoS One. 2013 Sep 9;8(9):e73845. doi: 10.1371/journal.pone.0073845 (PMC3767612; doi:10.1371/journal.pone.0073845)

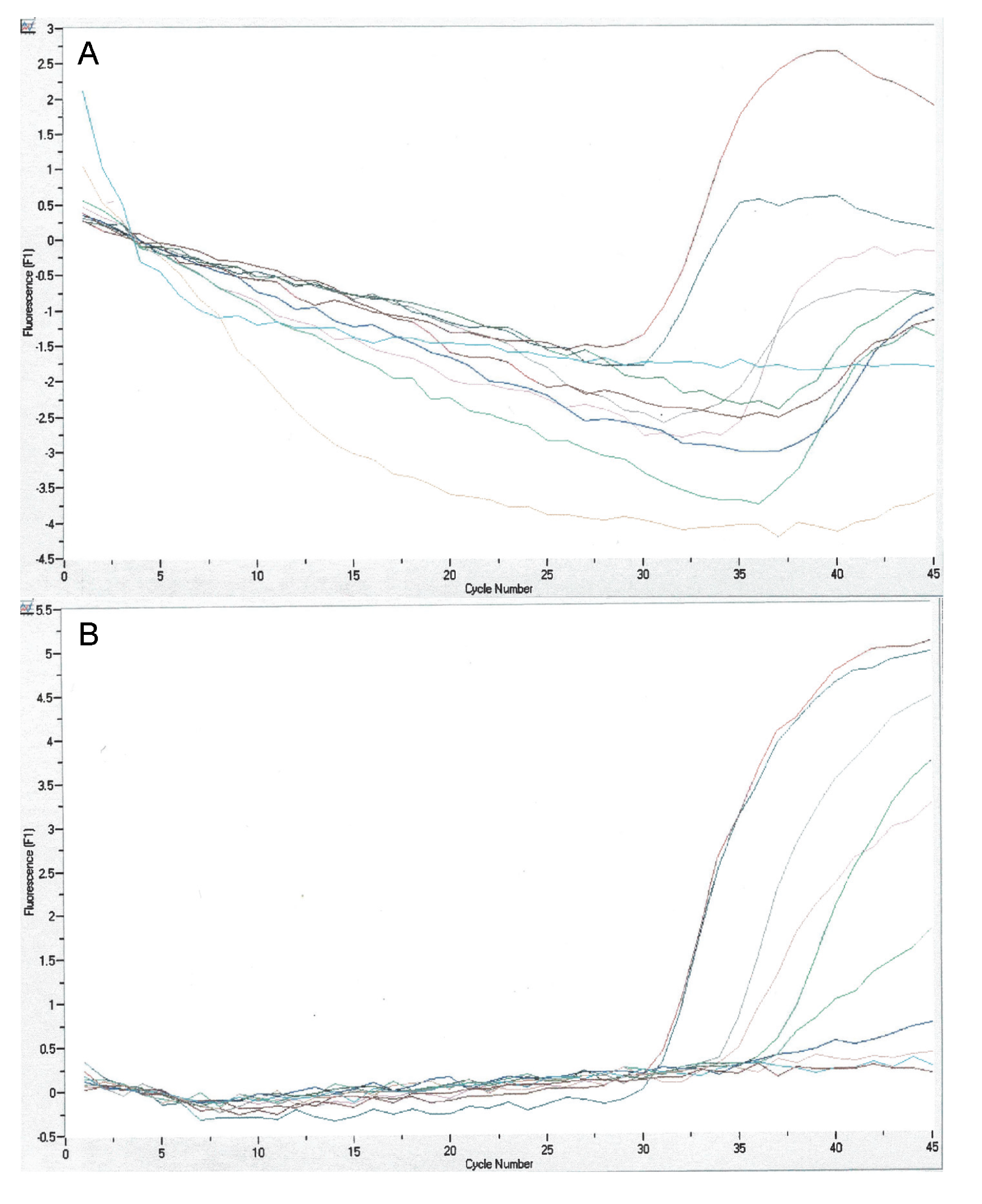

Supplement: Figure S1 — PCR kinetics and endpoint fluorescence comparison for Phire Hot Start and STRboost plus Phire Hot Start Polymerase. Both master mixes were tested with serial dilutions of DNA in 0.5% whole blood. On the y-axis (fluorescence), the range is -4.5 to 3 with increments of 0.5. The x-axis (cycle number) is 0 to 45 with increments of 5. (A) Phire Hot Start DNA polymerase master mix generated less than ideal real-time PCR kinetics and endpoint fluorescence. (B) When Phire Hot Start Polymerase was used with STRboost, the real-time PCR kinetics and endpoint fluorescence was greatly improved. The y-axis (fluorescence) ranges from -0.5 to 5.5 with increments of 0.5. The x-axis (cycle number) is 0 to 45 with increments of 5. (TIF) [file pone.0073845.s001.tif]
